# Methods for testing solubility of hydraulic calcium silicate cements for root-end filling

**DOI:** 10.1038/s41598-022-11031-z

**Published:** 2022-05-02

**Authors:** J. Camilleri, C. Wang, S. Kandhari, J. Heran, R. M. Shelton

**Affiliations:** School of Dentistry, Institute of Clinical Sciences, College of Medical and Dental Sciences, University of Birmingham, 5, Mill Pool Way, Edgbston, Birmingham, B5 7EG UK

**Keywords:** Calcium-based cement, Dental biomaterials, Mineral trioxide aggregate, Biomaterials, Biomedical materials

## Abstract

Regulatory testing of hydraulic cements used in dentistry and standard test methods for root-end filling materials do not exist. The aim of this study was to identify a simple, reproducible method for testing the solubility of materials that set with water (hydraulic) used as root-end filling materials in dentistry. Commercial and prototype hydraulic cements were characterized by scanning electron microscopy and X-ray diffraction analyses and their solubilities were determined using ISO 6876; 2012 standard, a modified ISO 6876 method with media alternative to water and a new method measuring the percentage mass loss and volume change of materials (micro-CT method) from a single surface exposed to three solutions. The solubility testing was performed by three operators to enable an intra-laboratory comparison. The solubility data obtained from the two commercial and two prototype materials varied depending on the method used, with the ISO 6876 method identifying differences in solubility of the materials (p < 0.05) but when modified with alternative solutions, no differences were found (p > 0.05). The changes in solution thus effected the solubility of the tested materials. Inter-operator differences were observed with the weight changes determined from the new method indicating this method was not robust. The weight and volume assessments using the new method were not solution-dependent. The advantage of the proposed method compared with the ISO standard is its simplicity, enabling a number of tests to be performed on the same set of samples that also more closely mimics the clinical environment.

## Introduction

Hydraulic calcium silicate cements have been introduced in dentistry specifically to be used as root-end filling materials^1^. The first product was composed of a mixture of Portland cement and bismuth oxide, which was marketed as “mineral trioxide aggregate” (MTA)^2,3^. The use of Portland cement arose from its setting with water^2,3^ with the tricalcium silicate phase forming calcium hydroxide on setting^4–6^. Hydraulic cements are used for a number of applications in endodontics^7^. Despite the widespread use of these materials in clinical dentistry, no specification for standard testing of hydraulic cements and no standards explicitly for testing of root-end filling materials are available. Most researchers use standards that were developed for testing other materials such as glass ionomers (ISO 9917-1; 2007)^8^, root canal sealers that are not hydraulic (ISO 6876; 2012)^9^ and resin-based materials (ISO 4049; 2019)^10^.

ISO test methods are developed to be sufficiently discriminatory but easily reproduced methods, using a thorough consensus process. Standards are produced for many different products and services, and may be created for company, national, regional or global application. Dental materials are classified as medical devices and their certification for clinical use requires compliance to specific standards with most standards for dental materials having been harmonized (EN 1641; 2009)^11^. The manufacturer/importer is responsible for its products and is potentially liable for damages. Furthermore, such standards are also used by a number of researchers as a tool to test materials.

Hydraulic calcium silicate cements used in endodontics are tested following the ISO 6876 standard^9^, which is the only standard dedicated to endodontic materials for flow, film thickness, setting time, radiopacity and solubility. This standard is designed to test sealer cements used in root canal obturation, thus the norms set for the materials may not be appropriate for root-end filling materials, which are also used in endodontics. Furthermore, hydraulic cements are different to other endodontic materials. The calcium hydroxide released after setting interacts with the environment the material is placed in. In contact with tooth structure, tissue fluids and blood, calcium phosphate or calcium carbonate is deposited on the material surface^12–14^. These interactions modify the solubility of tricalcium silicate-based materials, which has been reported to be higher^15–19^ than the 3 weight percent suggested in ISO 6876; 2012^9^. Solubility has been shown to be dependent on the immersion medium^20^. In alternative media simulating the clinical environment, which contain salts, the solubility of hydraulic cement sealers was higher than when water was used as immersing solution^20^.

The aims of the present research were to characterize 2 commercial and 2 experimental materials to assess their composition and microstructure and measure the solubility using ISO 6876; 2012 method using water compared with physiologically relevant solutions containing salts and proteins, which modify the material surface chemistry. The material chemistry and the interaction with the environment may influence the material solubility. In addition a micro-CT method was assessed for testing the solubility and volume change of such materials, and determine the reproducibility of the micro-CT proposed by intra-laboratory comparisons. The new method was intended to improve clinical representation of solubility whilst also being cheap, easy and reproducible. The null hypotheses were that the changes in solution did not affect the solubility of the materials tested, the testing undertaken was not robust and inter-operator variability existed.

## Methods

Two commercial hydraulic calcium silicate-based dental materials were tested (Biodentine; Septodont, Saint Maur des Fosses, France; MTA Angelus; Angelus, Londrina, Brazil) together with two experimental tricalcium silicate-based materials including: tricalcium silicate (TCS; Mineral Research Processing, Meyzieau, France), tricalcium silicate containing 20% zirconium dioxide (TCS-ZrO; Sigma-Aldrich, Gillingham, U.K.) as a radiopacifier. The TCS and radiopacified TCS were tested to assess the effect of the additives in the commercial materials.

The commercial materials were mixed according to the respective manufacturer’s instructions whilst the TCS-based prototypes were mixed with water at a water/powder ratio (by mass) of 0.35 for both the TCS and TCS-ZrO. Test samples 10 mm in diameter and 2 mm thick were prepared of each material type and were allowed to set in a humid environment in an incubator at 37 °C for 24 h. The end of setting was verified when an a final set Gilmore needle 1.06 mm in diameter and weighing 453.6 g (Impact Test Equipment, Stevenston, UK) failed to leave a mark on the material surface. Solubility was determined after immersion in either water, Hank’s balanced salt solution (HBSS; Sigma Aldrich, Gillingham, UK) or HBSS containing 10% fetal calf serum (Sigma-Aldrich, Gillingham, UK).

### Materials characterization

The material microstructure and chemical composition were assessed using scanning electron microscopy, energy dispersive spectroscopy and X-ray diffraction (XRD) analysis immediately after setting without any immersion or storage in media.

For scanning electron microscopy, samples were embedded in a cold-cure epoxy resin (Epoxy-fix; Struers, Ballerup, Denmark) and the surfaces were polished using an automatic polishing machine (Buhler, Lake Buff, IL, USA) using diamond discs (MD Piano; Struers, Ballerup, Denmark) under water coolant using 250, 500 and 1200 grit followed by polishing cloths MD Largo, MD Dac and MD Nap; (Struers, Ballerup, Denmark) using 9, 3 and 1 µm diamond impregnating polishing liquids. Polished samples were mounted on aluminium stubs (Agar Scientific, Stansted, UK) with double-sided carbon tape. An ultra-thin conductive gold coating (Emitech K550X; Ashford, UK) was sputtered on the polished surfaces, which were then viewed using the SEM (EVO MA10; Zeiss, Oberkochen, Germany) with an accelerating voltage of 20 kV and a working distance of 8.5 mm. The materials were then examined using back-scattered electrons to obtain elemental contrast at different magnifications and energy dispersive spectroscopy over an area was performed to assess the elemental distribution within samples.

For the X-ray diffraction (XRD) the materials were ground using an agate mortar and pestle to a fine powder. XRD was performed with a diffractometer (Bruker D8 Advance; Bruker, Billerica, MA, USA) with a CuKα radiation at 40 mA and 45 kV was set to rotate between 10° and 60° with a 0.02° 2θ step and a step time of 0.6 s. Phase identification was undertaken using a search-match software (DIFFRAC.EVA; Bruker, Billerca, MA, USA) using the ICDD database (International Centre for Diffraction Data, Newtown Square, PA, USA).

### Solubility assessment

The solubility assessment was performed using a method recommended by ISO 6876; 2012^9^ with water and also with variations in the liquid used. A new µCT-based method was also used by calculating the solubility by weight and volume changes after exposure to the solution. For both methods, the assessment was performed by 3 operators (CW, JH, SK) independently to have an intra-laboratory comparison. Each operator prepared and tested their own specimens using the same batches of materials and same equipment.

#### ISO 6876 method

The materials were mixed as indicated in the materials section. Six specimens measuring 20 mm in diameter and 1.5 mm high were prepared for each material type and immersion medium. The materials were allowed to set for 24 h at 37 °C in 100% humidity before weighing to the nearest 0.001 g (TS400D, Ohaus, Florham Park, NJ, USA). The samples (n = 2) were placed in a shallow dish and 50 ± 1 mL of either deionized water as suggested by the ISO standard, or alternatively in HBSS or HBSS containing 10% fetal calf serum. The use of alternative solutions is a deviation from the ISO standard. The container was covered and allowed to stand for 24 h before transferring all contents to a second dish after filtering. The liquid was evaporated at 110 ± 2 °C until a constant mass was obtained and the containers were placed in a desiccator at room temperature to cool before weighing. The difference in mass of the dish before and after drying as the amount of material removed calculated as a percentage of the original combined mass of the two specimens expressed the material solubility in the different solutions. The experiment was repeated three times for each material and each solution and the experiments are undertaken by three operators working independently.

#### Micro-CT assessment

Perspex blocks measuring 20 × 20 mm were prepared containing a standard cavity 4 mm in diameter and 3 mm deep drilled in the centre of each block. All Perspex blocks were weighed and the mass recorded as M_0_. The four hydraulic calcium silicate materials were mixed and compacted into the cavities using a stainless-steel condenser and a microscope slide was then placed over the mould to ensure a flat sample surface. The materials were allowed to set at 37 °C for 24 h in 100% humidity. The materials and blocks were weighed again and mass recorded as M_1._ All the weights were taken to the accuracy of 0.0001 g. Six blocks were prepared for each material and each solution tested.

Microcomputed tomography was performed and images of samples were obtained using a µCT scanner (SkyScan 1172; Bruker, Billerca, MA, USA) at 70 kV and 142 µA in the presence of a 0.5 mm aluminium filter at ambient temperature (22 °C). A flat field correction was taken on the day, prior to scanning to correct for variations in the pixel sensitivity of the camera. Images were reconstructed using software (NRecon Version 1.4.0; Bruker, Billerca, MA, USA) with ring artifacts reduction of 13 and beam-hardening correction of 20%. The volume of the materials was determined (CTAn, Version 1.18.4.0+; Bruker, Billerca, MA, USA).

After the initial measurements, the blocks were then immersed in either 15 mL of water, or HBSS or HBSS + FCS at 37 °C. After 1 week the blocks and materials were retrieved and surface dried using a filter paper before weighing (recorded as M_2_). The volume of the materials was determined using µCT. All the measurements were undertaken by three operators to have intra-laboratory comparisons and changes in mass and volume were determined.

### Surface characterization and leachate analysis

Surface characterization was performed using SEM after the completion of the solubility assessment using the Perspex blocks. For SEM, the material surfaces were coated with gold and imaged using secondary electrons. Images were captured at 500, 100 and 2000 magnification to assess surface microstructural changes. The leachates collected from samples immersed in the different solutions were analysed for calcium, silicon, zirconium and tungsten with inductively coupled plasma mass spectroscopy (ICP-MS; Optima 8000, Perkin Elmer, Waltham, (MA) USA).

### Statistical analysis

Statistical analysis was performed by one operator using Predictive Analytics Software (PASW version 18; IBM, Armonk, NY, USA). One-way ANOVA was used to determine whether there were significant differences among data sets. The data was tested to ensure it was normally distributed and then with analysis of variance with p = 0.05, the Tukey post-hoc test was used.

## Results

### Material characterization

**Figure 1:**
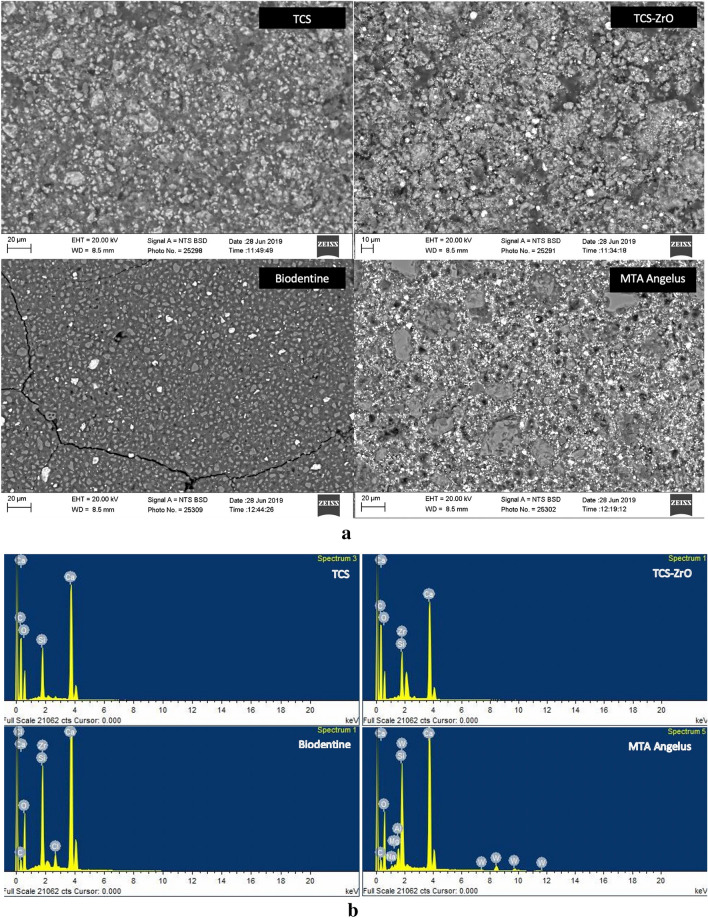

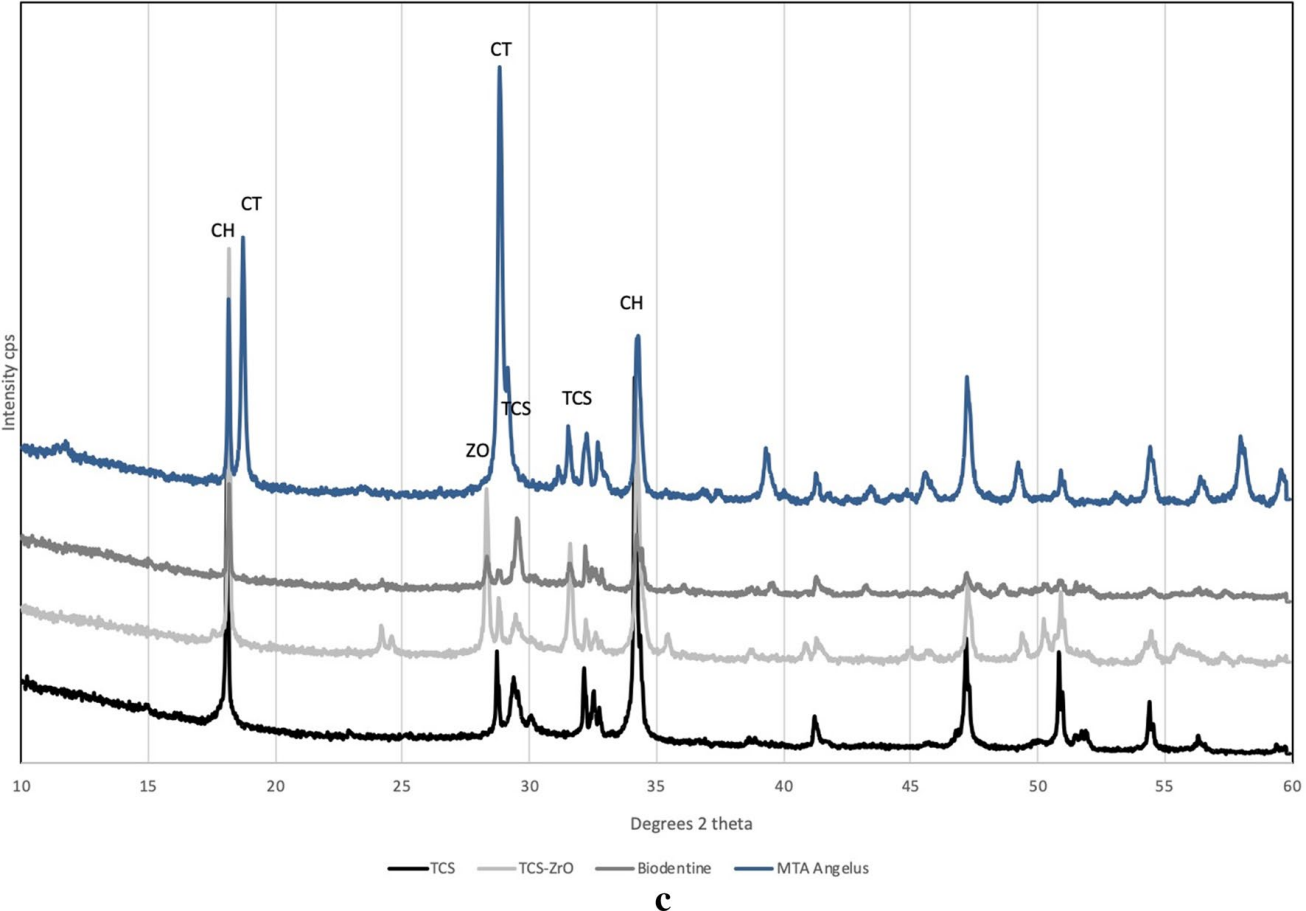
(**a**) Back scatter scanning electron micrographs of the test materials showing microstructural features indicating the different chemistry and microstructure (× 1 K magnification). The images show the particle sizing and radiopacifier loading with Biodentine having a smaller particle size when compared to the TCS-ZrO and MTA Angelus and less radiopacifier compared to all materials. (**b**) EDS area plots of the materials showing the elemental composition. All the materials showed peaks for calcium, silicon and oxygen. (**c**) XRD plot of the test materials showing the main phases present (*CH* calcium hydroxide, *CT* calcium tungstate, *TCS* tricalcium silicate, *ZO* zirconium oxide.

The scanning electron micrographs of the polished materials immediately after setting are shown in Fig. 1a. The MTA Angelus was composed of unhydrated cement particles, which were approximately 10–15 µm in diameter with the radio-opacifier clearly visible due to its high molecular mass as numerous bright electron dense particles. The Biodentine particle diameters were less than 5 µm and less radio-opacifier was present when compared with MTA Angelus.

The TCS particles were approximately 10 µm in diameter and the inclusion of zirconium oxide was evident in the micrograph due to its heavier atomic mass thus appearing as bright electron dense particles. The EDS analysis in Fig. 1b shows calcium, silicon and oxygen present in all the materials, chlorine in Biodentine and zirconium was identified in Biodentine and TCS-ZrO, whilst MTA Angelus also exhibited peaks for tungsten, magnesium, aluminium and sodium.

The XRD data is shown in Fig. 1c and all the materials exhibited tricalcium silicate peaks evident at 29° 2θ and 2 peaks in the 32° 2θ region. Calcium carbonate peaks were also identified in Biodentine with the main peak at 29.5° 2θ. Biodentine and the radio-opacified TCS also demonstrated peaks for zirconium oxide while the MTA Angelus contained calcium tungstate. All the materials demonstrated calcium hydroxide peaks at 18 and at 34° 2θ, which indicated that cement hydration had occurred during setting.

### Solubility assessment

#### ISO 6876 method

**Figure 2:**
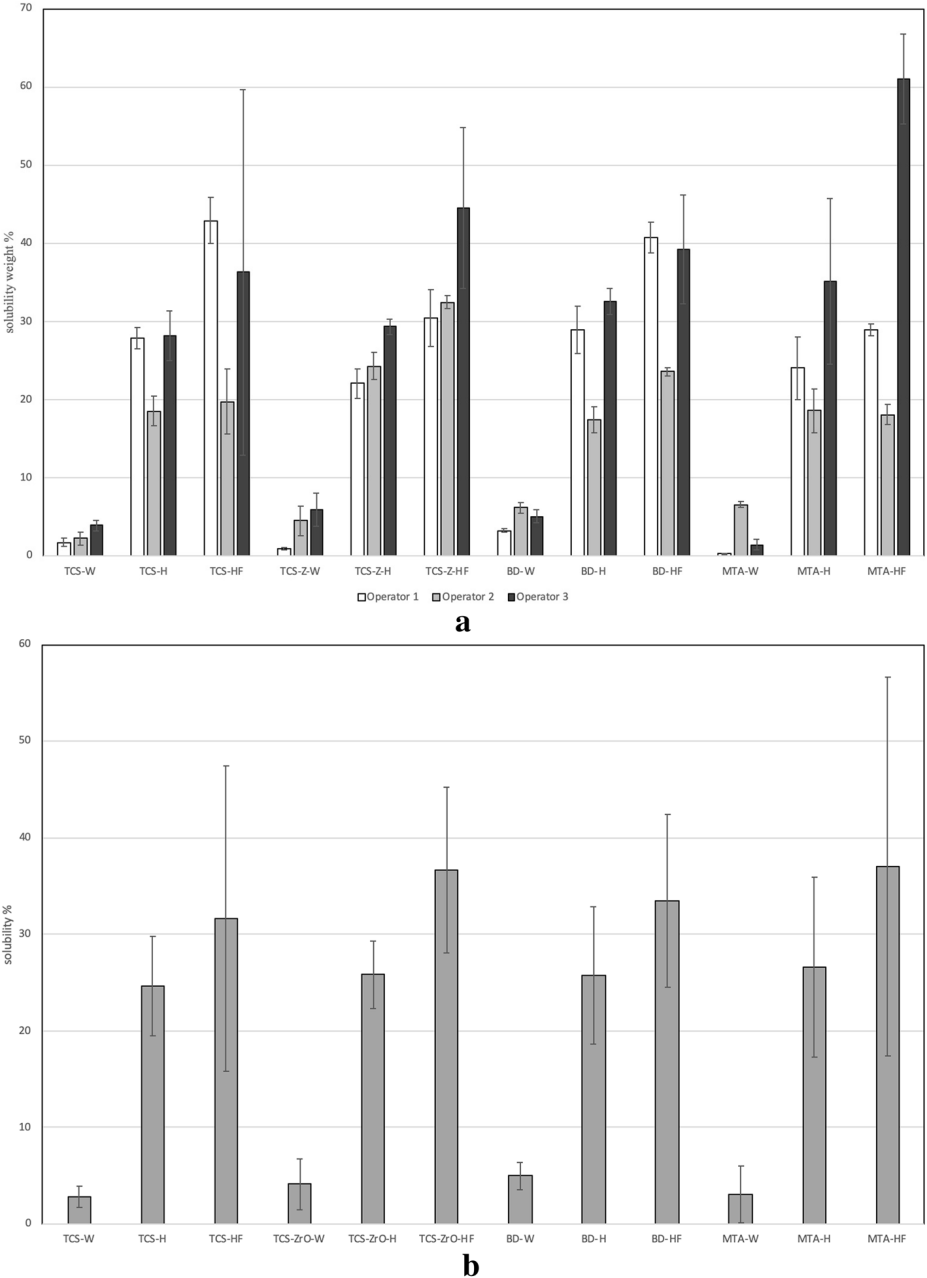
Mean percentage solubility of test materials tested according to ISO 6876; 2012 specifications using water as soaking solution, which is specified in the ISO 6876 method and alternative solutions, which are more clinically relevant. (**a**) Three sets of data of testing undertaken by different operators are shown indicating the dependability on the immersing liquid; (**b**) Pooled data for solubility testing of the materials (± SD).

The solubility of the materials after 24 h determined using the ISO 6876; 2012 method^9^ with different immersion solutions and the tests undertaken by different operators is shown in Fig. 2a. The intra-laboratory comparisons showed that the operators obtained similar results for all the tests carried out (p > 0.05) except when testing MTA Angelus in Hank’s with fetal calf serum (p < 0.0001), which indicated that the ISO 6876 method was repeatable.

The pooled data is shown in Fig. 2b. The solubility was dependent on the solution used for all materials tested with lower solubility shown in water (p < 0.001) when compared with HBSS and HBSS + FCS. Sample solubility in the latter two solutions showed some differences (p > 0.05). All materials exhibited similar solubility values (no statistically different data) when tested in the same immersion media (p > 0.05).

The solubility in HBSS and HBSS + FCS was higher than the 3% specified by the ISO standard^9^. However altering the liquid from water requires a variation from the standard and it is unlikely the limit of 3% is still applicable. The TCS-ZrO and both commercial materials also did not comply with the ISO specification and exhibited solubility higher than 3% in water.

#### MicroCT assessment

**Figure 3:**
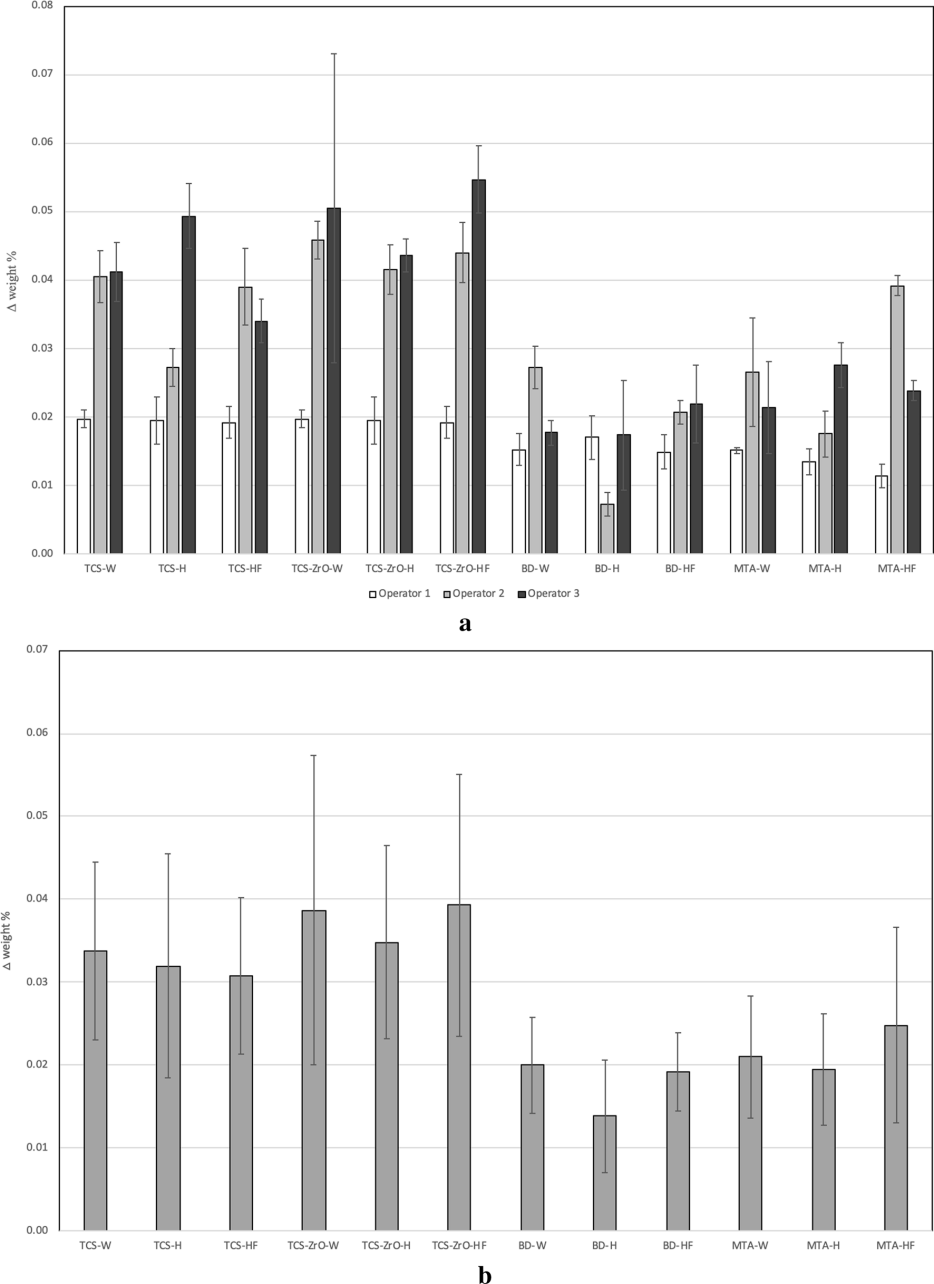

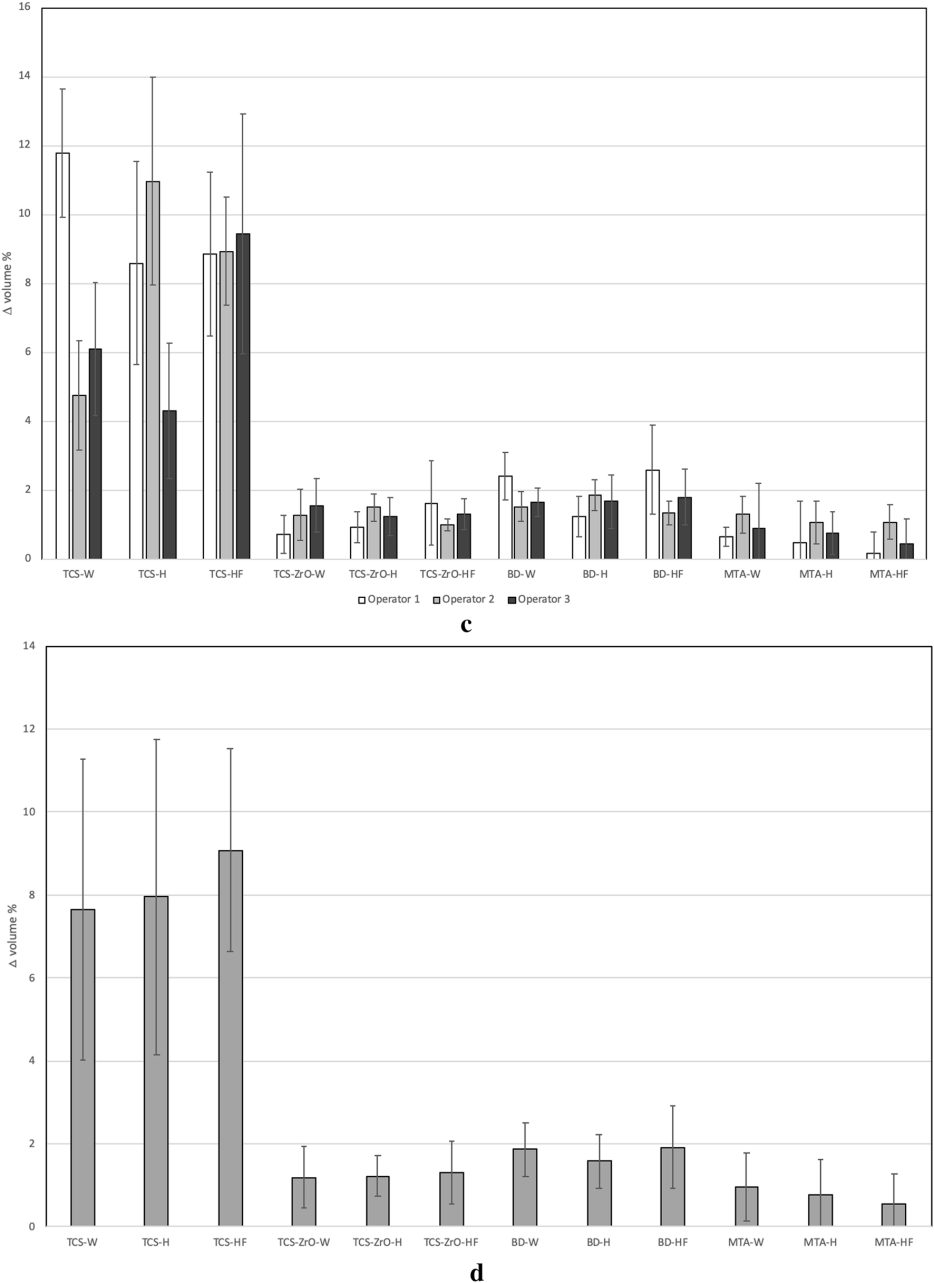
(**a**, **b**) Mass, (**c**, **d**) volume changes of the materials after 1 week in different soaking solutions showing (**a**) the variation of the data obtained by the different operators for the changes in mass and (**b**) pooled data; (**c**) there was less inter-operator variation for the volume determinations and (**d**) indicating the higher volume changes for tricalcium silicate compared to the other materials. All testing independent of the solution used.

The µCT assessment solubility determination involved a multiparameter assessment of the samples. Mass changes for the individual operator data are shown in Fig. 3a and the pooled data in Fig. 3b. The volume determinations are shown in Fig. 3c and d respectively. Variation among the operators in the weight assessments (p < 0.001) was present indicating that the method was not robust nor repeatable (Fig. 3a). The pooled data (Fig. 3b) showed a higher percentage weight gain exhibited by the TCS and TCS-ZrO compared with the commercial materials in all solutions used (p < 0.001). Thus, this method identified differences in material behaviour irrespective of the solution.

The volume change assessment (Fig. 3c) showed no differences irrespective of the operator (p = 1.000) except for the TCS analysis. The pooled data (Fig. 3d) showed that TCS exhibited a higher volume change than the materials including additives (p < 0.001), which indicated that the pure cement was more susceptible to volume change than materials incorporating additives. All materials including additives exhibited volume changes of less than 2% and were not influenced by the immersion media used (p > 0.05).

### Surface characterization and leachate analysis

**Figure 4:**
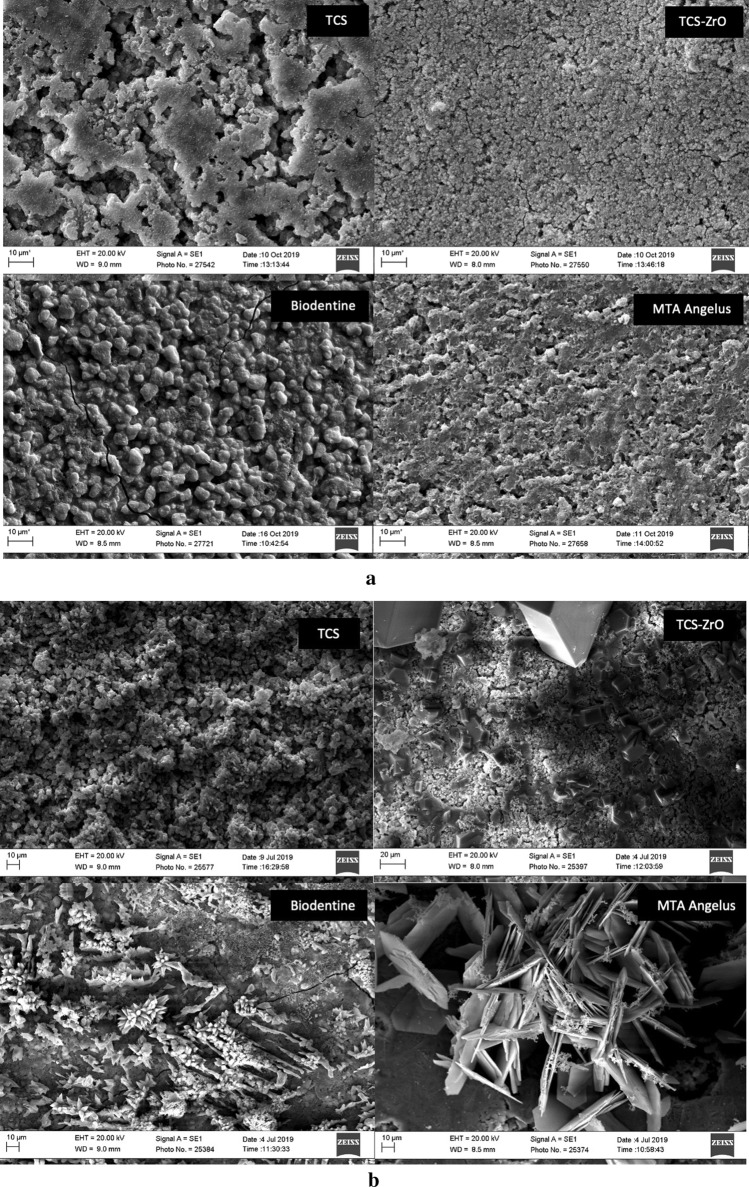

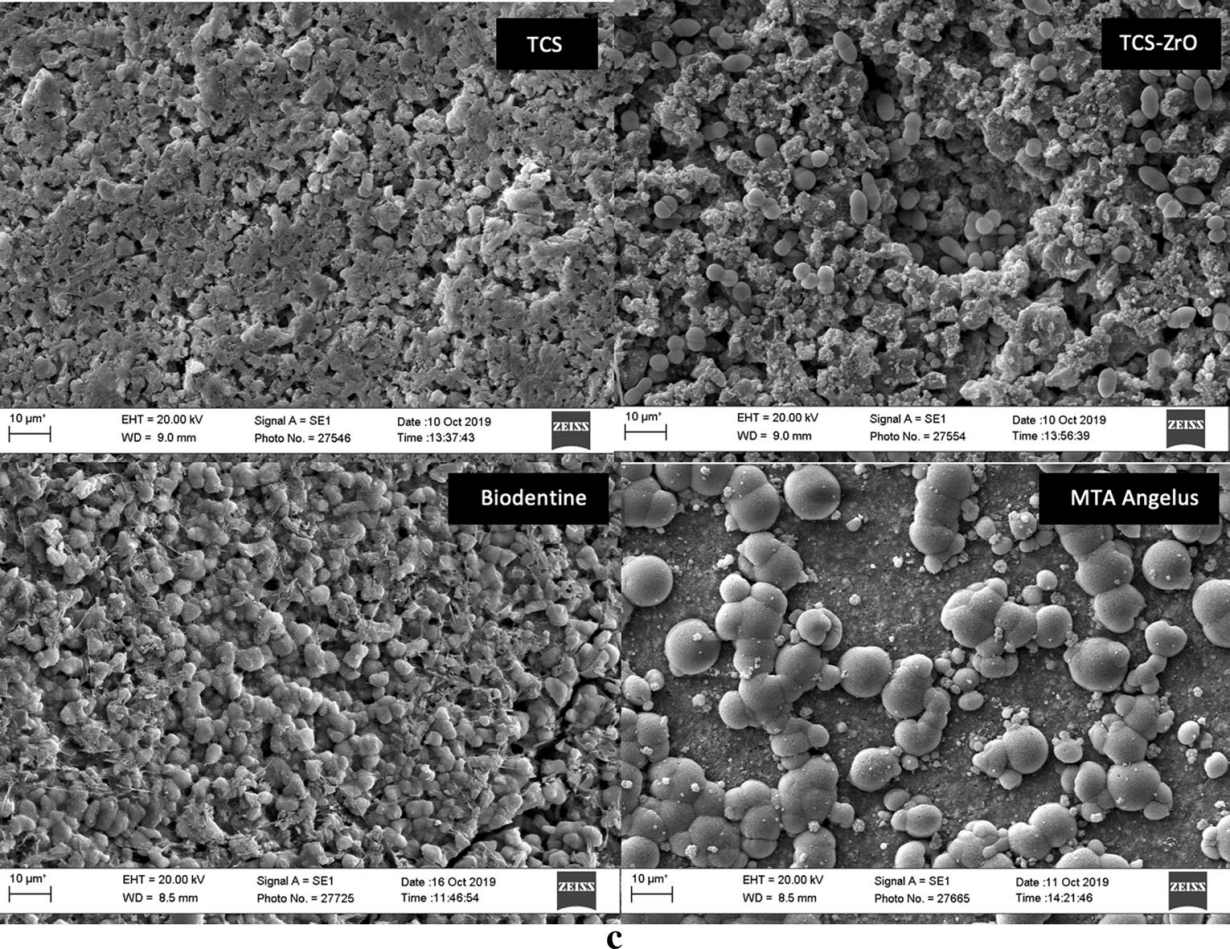
Secondary electron scanning electron micrographs showing surface microstructure of all test materials in contact with (**a**) water, (**b**) HBSS, (**c**) HBSS + FCS. For all solutions various surface deposits were observed with generalized discreet crystals observed over the surface of all materials in water, calcium hydroxide crystals (hexagonal) and calcium phosphate (spheres) in HBSS and discrete round crystals of calcium carbonate for the HBSS + FCS.

Scanning electron micrographs in Fig. 4a–c show the surface microstructure of the four materials in the three immersion media. Surface deposits were observed on all materials in all solutions, which indicated that reactions took place at the surface. The interaction for each material with the solutions appeared to differ; the deposits ranged from deposition of calcium hydroxide (plate-like crystals) observed in both water and HBSS, to globular crystals synonymous with calcium carbonate deposits mostly seen in the HBSS + FCS group.

**Figure 5:**
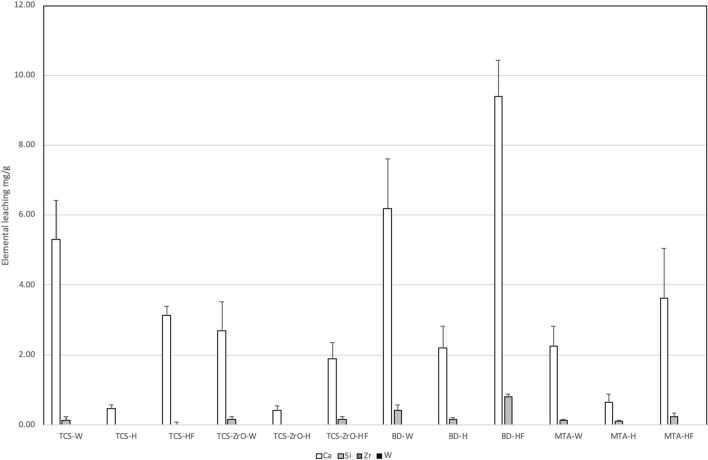
Elemental leaching in different solutions shown by the test materials showing a variation in the leaching of calcium and silicon observed by all materials while zirconium was negligible.

The ICP data is in Fig. 5. The ions varied according to the solution used, although zirconium and tungsten were not detected in any of the solutions. All materials leached calcium ions with the HBSS showing the lowest values. Materials placed in HBSS + FCS exhibited higher levels of calcium in solution than identified in HBSS.

## Discussion

The null hypothesis that the changes in solution did not affect the solubility of the materials tested was rejected. The null hypotheses that the testing undertaken was not robust and inter-operator variability existed were partially rejected as the ISO 6876; 2012 method and volumetric assessments of the materials that included additives showed no inter-operator variation.

The data on solubility of MTA and hydraulic calcium silicate cements is diverse with some studies showing negligible solubility for MTA and root-end filling materials^21–23^ ranging to considerably higher values (22% to 31%) also for MTA^24,25^ compared with the ISO 6876; 2012 standard. Material solubility for MTA was found to depend on the water/powder ratio in previous studies^24,25^ as the addition of more water to MTA increased its solubility^24–26^. Changes to the water to powder ratio modify material properties such as calcium ion release and flow, particularly if there are additives in the mixture such as radiopacifiers or nucleating agents as the calcium carbonate in the Biodentine, and the changes to the effective water to powder ratio are not taken into consideration^27^. In the current study two commercial hydraulic calcium silicates were selected with Biodentine including a water-soluble polymer that allows use of a low powder to liquid ratio.

Differences in the literature for solubility testing may be attributed to variations in the methodology used, in particular in  methods where weight changes are measured over time with continuous wetting and drying cycles. If the materials are dried in air, the calcium hydroxide will react with the atmospheric carbon dioxide leading to deposition of calcium carbonate on the surface. The ISO 6876; 2012^9^ methodology suggests the use of a desiccator but this does not limit the interaction of atmospheric carbon dioxide with the sample leading to carbonation. Such deposition could have increased the sample mass, possibly accounting for the negligible test material solubility identified in a number of studies^21–23^. Unfortunately, it is difficult to compare the solubility of the same materials in different studies without standardised methodology. In some studies the solubility has been measured by monitoring the sample mass over a period of time for root-end filling materials^15,21^ and root canal sealers^18,20^. Changes in sample mass as a measure of solubility differs from the ISO 6876; 2012^9^ method where solubility should be measured according to the difference in mass of the immersion solution rather than the sample itself. In fact, a study comparing the two methods generated different data from identical sealer samples^20^. Other methods have included the measurement of solubility from the mass of the leachate from root canal sealers over a period of time accompanied by leachate analysis^17^ and the use of microcomputed tomography to assess volumetric changes of root canal sealers^16^. In this latter method there were still variations in the methodology adopted, using root-end fillings in acrylic teeth immersed in water, whilst human teeth were placed in phosphate-buffered saline^16^. Another method evaluated the volumetric changes of root-end filling materials compared with the volume of a standardized cavity filled with a radio-opaque material without using a solution^15^. The change in methodology for solubility requires robust testing and also needs to take into account material chemistry, as not all tests are suitable for hydraulic cements arising from potential interactions within the experimental environment.

Variations in the immersion solution has been used despite ISO 6876; 2012^9^ specifying the use of water. Water is recommended as this is easy to standardize; the HBSS and HBSS + FCS are less so. Furthermore until the use of hydraulic cements all endodontic sealers were inert thus did not interact with the liquids they were placed in. Hydraulic cements interact with the environment and thus water does not represent a suitable immersing solution to test such materials since tissue fluids contain a number of ions and proteins. In fact, the use of solutions that contain salts such as HBSS and DMEM identified different solubility values for hydraulic cement sealers when compared with the solubility in water^20^. HBSS and DMEM have been proposed as their composition is similar to the physiological solution thus mimicking the clinical situation for root-end filling materials. The dependence of solubility on the immersing solution when using the ISO 6876; 2012 method has also been shown in the current study. The current study also indicated that not only does the ISO 6876; 2012 method depend on the solution used but also in alternative solutions, the method cannot differentiate among the materials tested. While in water a difference was observed, the changes in alterative solutions were not evident due to the changes occurring over the material surfaces as shown in the scanning electron micrographs and leachate assessment. This indicated that the ISO 6876; 2012 method is not suitable to test hydraulic cements as water is not a suitable immersing solution and also the use of alternative more clinically relevant solutions does not allow differentiation among the materials.

The ISO 6876; 2012 uses 2 specimens per test. Scientifically the use of n = 2 raises concerns as the low number will not permit adequate statistical analysis. The use of two specimens per solution may be suggested to have a large surface area available through which the material solubility can occur. If the method is being used for research purposes, it is important to run test replicates as undertaken in the current study to ensure test repeatability and the possibility to run accurate statistical analysis.

The µCT method used to assess the solubility of root-end filling materials required standardized blocks, which were relatively cheap and easy to produce. The use of the blocks rather than material discs rendered the scenario more similar to the clinical situation for root-end filling materials where only one surface was exposed to the dissolution environment; this cannot be undertaken with the material discs where multiple surfaces are exposed to the solution and the solubility measurement depends on the volume of the disc, which is not clinically relevant. Furthermore, using the same specimen allowed a number of different tests to be undertaken. These included mass measurements as in previous studies for both root canal sealers^18^ and root end filling/pulp capping materials^15,19,21^ as well as volumetric changes with some modifications to previous studies for root canal sealers^16,18^ and root-end filling materials^15,27^.

Material characterization and also surface characterization was performed in the current study. Material characterization should be a requirement for all material testing^28^ as knowledge of material chemistry will facilitate the interpretation of physical test data. In the current study the materials were tested in both a wet/saturated and surface dry state, where only the superficial moisture was removed rather than desiccated as typically performed in solubility experiments.

Intra-laboratory comparisons were conducted to identify the repeatability of the method used. Both the ISO 6876; 2012 method and the volumetric analysis showed little inter-operator variation, thus are recommended as reliable methods of analyses. Regardless of the lack of inter-operator variation, the solubility determined using the ISO 6876; 2012 method was immersion solution dependent. This method uses large volumes of immersing liquid that when evaporated left crystals, which changed the weight of the residue and altered the solubility values. Water is not a suitable or representative immersing liquid for hydraulic calcium silicate cements as these materials interact with complex tissue fluids in vivo^29^.

The µCT method proposed used sample mass and volume changes to assess the material solubility. The analysis can be conducted over different periods of time when using this method thus allowing longitudinal assessment as the test is non-destructive. The data for the mass and volumetric changes were not affected by the solutions used but varied depending on sample material chemistry as the materials including additives exhibited lower volumetric and weight changes than the TCS. The only disadvantage of this method was that the weight assessment showed some inter-operator variation possibly due to the sample size and the fact that the wet/saturated and surface dry states cannot be absolute conditions. Another disadvantage of the alternative method was that the equipment used for the volume assessment was specialized and not necessarily widely available.

The measurement of leaching in solution should not be confused with solubility nor be used to replace solubility testing. Solubility measures the removal of solid material rather than the chemical displacement of ions in solution. The materials leached calcium in solution and the leaching pattern varied depending on the solution used. Synthetic tissue fluids may not necessarily replicate the in vivo conditions and this limitation has previously been reported^30^. However, the presence of proteins in solution is more representative of the in vivo environment and it was unusual that this did not influence the solubility nor leaching patterns to a measurable extent as has previously been demonstrated on bioactive surfaces^31–33^. The crystalline deposits were observed on all materials in all solutions indicating the surface reactivity of the hydraulic cements.

Although the use of volumetric changes in a physiological protein-based solution using standard blocks that allows longitudinal testing and also material characterization and other tests to be undertaken is considered to be the best way to assess solubility of root-end filling materials, the equipment required to undertake these experiments is not readily available and also involves considerable expense. Furthermore the test is complex and requires operator training.

## Conclusions

An alternative method to the ISO 6876; 2012 using µCT measurements on standardized specimens with a single surface exposed to solution has been proposed to measure the solubility of hydraulic calcium silicate cements. This test may be more reproducible and clinically relevant for dental root-end filling materials. The alternative method allows longitudinal testing of parameters weight and volume change.
